# Barriers and enablers of work–life balance among regulatory affairs pharmacists working remotely in Gauteng, South Africa: A qualitative study

**DOI:** 10.4102/hsag.v31i0.3184

**Published:** 2026-10-02

**Authors:** Kgabo M. Sekgalakane, Tintswalo M. Hlungwani, Ané Orchard

**Affiliations:** 1 Department of Pharmacy and Pharmacology, Faculty of Health Sciences, University of the Witwatersrand, Johannesburg, South Africa; 2 School of Public Health, Faculty of Health Sciences, University of the Witwatersrand, Johannesburg, South Africa

**Keywords:** mental health, productivity, regulatory affairs pharmacist, remote work, work–life balance

## Abstract

**Background:**

Regulatory Affairs (RA) pharmacists in South Africa transitioned to remote work during the COVID-19 pandemic and continued post-restrictions. Limited research on their work–life balance (WLB) experiences hinders supportive workplace policy development and understanding of work–family conflict dynamics to inform sustainable remote work practices.

**Aim:**

This study aimed to explore the WLB experiences of RA pharmacists following the easing of COVID-19 restrictions.

**Setting:**

The study was conducted in Gauteng province, South Africa.

**Methods:**

An exploratory qualitative design used semi-structured interviews guided by Bronfenbrenner’s framework and the Work–Life Harmony model. Data from 17 participants were analysed thematically using inductive and deductive approaches.

**Results:**

The WLB barriers and enablers emerged across socio-ecological levels. The microsystem included boundary challenges, overwork, isolation and adaptive routines. The mesosystem reflected family support, work–life enrichment and competing demands. Exosystem factors, including micromanagement, inconsistent policy implementation and limited resources, undermined flexibility and autonomy, while supportive management and peer collaboration enhanced WLB. Macrosystem regulatory demands, time-zone pressures and an output-driven culture extended working hours. The chronosystem reflected evolving work arrangements and boundary-setting.

**Conclusion:**

Remote and hybrid work increased flexibility, family engagement and reduced commuting for RA pharmacists. However, blurred boundaries, overtime, constant availability, micromanagement, inconsistent policies, staffing shortages and poor support undermined autonomy and mental health.

**Contribution:**

The study provides unique insights into WLB barriers and enablers among RA pharmacists, informing management strategies and psychological support for remote work optimisation.

## Introduction

Remote work, defined as working from home or outside the conventional office environment using internet connectivity, has been explored since the early 1970s (Allen, Golden & Shockley 2015). Global studies on remote work show that working from home has both positive and negative outcomes on work–life balance (WLB) and well-being (Felstead & Henseke 2017; Stasiuk-Piekarska 2021).

Work–life balance is described as the state of equilibrium between an individual’s work role demands, intrinsic factors such as emotional regulation, and other everyday life roles (Hasan et al. 2020). This does not imply an equal distribution between roles but rather an absence of unmanageable interference between them (McMillan, Morris & Atchley 2011). Well-being can be characterised as the positive psychological experiences of individuals, closely related to their overall quality of life. It encompasses emotional, mental and physical factors that contribute to subjective happiness (Stasiuk-Piekarska 2021).

During the coronavirus disease 2019 (COVID-19) pandemic, remote and hybrid working models were heightened globally because of mobility restrictions aimed at reducing virus transmission and facilitating economic continuity (Naidu & Hoque 2022; Rajagopaul et al. 2020; Telukdarie, Munsamy & Mohlala 2020). In South Africa, COVID-19 restrictions were implemented on 15th March 2020 following the declaration of a State of National Disaster (Department of Co-operative Governance and Traditional Affairs [South Africa] 2020). The COVID-19 pandemic presented unique obstacles, as people experienced feelings of loneliness and isolation, particularly those who lived alone and were separated from their families (Kangas, Pensar & Rousi 2023). Furthermore, remote or isolated work setups can harm organisational collaborative culture, potentially heightening employee stress and negatively impacting retention (Henneman, Thornby & Bookstaver 2022; Prescott, Kennedy & DeLuca 2022). Post-easing of the COVID-19 restrictions, the influence of isolation on the mental health of individuals remains evident, as hybrid and remote work practices continue to be commonplace. Consequently, workers in these work arrangements continue to experience heightened feelings of loneliness, reduced mental stimulation and disconnection from their communities (Kangas et al. 2023; O’Hare et al. 2024). Addressing isolation by focusing on employees’ well-being and WLB can contribute to a more nuanced understanding of the remote working model and facilitate business continuity and productivity in this sector.

In the health sector, literature on the experience of academic pharmacists teaching remotely during the pandemic is well-documented (Gallegos et al. 2022; Henneman et al. 2022; Prescott et al. 2022). A study showed that 85% of pharmacy faculty in the United States reported no change in productivity, while 80% reported no change in effectiveness and an improved WLB. Remote work introduced limitations related to accurate screening and diagnosing, an increased likelihood of diagnostic errors and digital data overload (Oleksa-Marewska & Tokar 2022). In the community pharmacy sector, data overload can be managed through the use of streamlined and integrated procedures and systems (Hettinger et al. 2024), whereas, in the industrial pharmacy sector, data overload can be addressed through the implementation of Regulatory Harmonisation and Reliance (World Health Organization [WHO] 2021).

Harmonisation refers to the standardisation of regulatory review procedures and standards, guidelines and implementation of International Council on Harmonisation of Technical Requirements for Pharmaceuticals for Human Use (ICH) standards (ICH M2 Expert Working Group 2008; WHO 2021). Reliance refers to a regulatory approach whereby one authority considers and uses the scientific assessments or decisions of another trusted regulatory body when making its own determinations (WHO 2021). The lack of the aforementioned potentiates additional workload pressure for regulatory affairs (RA) Pharmacists because of duplicated efforts when submitting multiple dossiers across different African nations where medicine registration timelines, technical requirements, digitalisation and medicine access enablement strategies differ.

The impact of delayed harmonisation and effective implementation of reliance across Africa is evident at the National Regulatory Authority (NRA) level. These factors contribute to delays in the review of new medicine registration dossiers and variation applications, resulting in backlogs and delayed provision of essential medicines to the public, as was evident at the South African Health Products Regulatory Authority (SAHPRA), where 16 000 backlog applications were inherited from the Medicines Control Council (MCC) in 2018 (Danks et al. 2023). Contrastingly, in the developed Global North, the European Union-initiated harmonisation strategies have been in place since the 1980s (European Medicines Agency 2025). They are further supported through the adoption of the electronic Common Technical Document (eCTD) in line with the ICH, and Mutual Recognition Agreements (MRA) with different NRAs. Therefore, concepts such as reliance and regulatory harmonisation are well-documented in these regions.

The role of the RA Pharmacist is dynamic, such that they could be permanently employed by a pharmaceutical company in South Africa yet still work remotely across different territories in Africa where the company is authorised to market medicines. Flexible hybrid working arrangements existed for RA Pharmacists before the COVID-19 pandemic and continue post-easing of the COVID-19 restrictions. This benefits the African continent as it adds to the efforts to actualise Sustainable Development Goal 3 (good health and well-being) by increasing access for patients to safe, efficacious and quality medicines, where the triple burden of disease remains evident and prominent (United Nations Department of Economic and Social Affairs (DESA) 2025). However, challenges with working across multiple territories include time zone considerations, which could force the South African RA Pharmacist to work outside core working hours (Chauvin, Choudhury & Fang 2024; Kangas et al. 2023). Additionally, delayed harmonisation and ineffective implementation of reliance across the African continent can lead to extended working hours. In South Africa, factors such as load-shedding delay digitisation and further add to the work pressures due to the sometimes-unpredictable load-shedding schedules. The available literature addresses aspects such as load shedding, delayed harmonisation and digitisation and their varying impact on the pharmaceutical sector (Danks et al. 2023; Department of Health 2022). However, the influence of all these combined factors on the overall WLB experience and productivity of RA Pharmacists, a core stakeholder in the pharmaceutical sector, remains overlooked and undocumented.

Despite global evidence on remote work, there remains a paucity of literature examining the WLB experiences of RA pharmacists in South Africa. As long as these experiences remain undocumented, remote work policies may fail to adequately support employee well-being and sustainable productivity. This study therefore explores the barriers and enablers influencing WLB among RA pharmacists working remotely in Gauteng.

### Theoretical and conceptual framework

The Work–Life Harmony theoretical model used in this study describes themes of WLB, Work–Life Conflict (WLC) and Work–Life enrichment, which can have cross-domain effects (Hasan et al. 2020). The cross-domain effect describes how themes can influence others positively or negatively if there is a heightened imbalance in one area (Hasan et al. 2020). This aligns with Bronfenbrenner’s 1979 multi-factored socio-ecological conceptual framework, which describes how human development and behaviours are determined by interactions between individuals and their environments (Bronfenbrenner 1979). These frameworks complement each other as they demonstrate the influence and association between the interactions of the individual and the environment (Online Appendix Table 1-A1 and Figure 1-A1). Bronfenbrenner’s model looks at external, systemic factors such as organisational policies and family dynamics, while the Work–Life Harmony model focuses on internal, psychological outcomes (e.g. WLC and enrichment). The Work–Life Harmony model can be located within Bronfenbrenner’s framework and was used to describe the remote working experience of RA Pharmacists in Gauteng by focusing on factors such as cognition, behaviour, well-being and environmental interactions.

## Research methods and design

### Study design

An explorative qualitative and descriptive research design method was used, guided by Bronfenbrenner’s conceptual framework and the Work–Life Harmony theoretical framework. The explorative qualitative research design was chosen as it is appropriate for understanding complex social phenomena like WLB. Qualitative research is suited to provide a broader understanding when uncovering the depths of personal experiences, nuances in perceptions and the complexities of the impacts of remote work that might be missed in quantitative research (Creswell 2013).

### Setting

The study was conducted in Johannesburg, Gauteng province of South Africa. Johannesburg was selected as the study setting as it is the primary commercial and corporate hub for pharmaceutical companies and RA professionals, enabling access to participants with diverse roles, experience levels and organisational contexts within the sector. The study site was informed by the database of inspected, registered and approved Applicants by SAHPRA. The database is updated and maintained by the Health Authority and can be accessed on the following website: https://www.sahpra.org.za/approved-licences/ (SAHPRA 2024).

### Study population and sampling strategy

#### Participant recruitment

The study population consisted of RA Pharmacists based in Johannesburg, South Africa working remotely or with experience working in a hybrid system post-easing of the COVID-19 restrictions. The researcher sent an open call for participation via LinkedIn. A total of 18 individuals expressed interest in the study. One participant withdrew prior to the interview, resulting in a final sample of 17 participants. Data saturation was assessed iteratively during analysis and was reached at 17 participants, as no new themes emerged. Using social media and snowball sampling is an effective technique to identify participants within a niche group. This ensures a broader pool of respondents that are often difficult to reach through other traditional recruitment strategies.

#### Sampling and sample size

Sample size in qualitative research is often guided by data saturation. Saturation or ‘data adequacy’ describes the point at which repeated detailed description is achieved, and no additional codes and themes are gathered from additional participants (Morse 1995; Rahimi & Khatooni 2024). Bernard (2018) suggests a sample size of 10–20 is sufficient to identify critical issues and provide rich data in qualitative studies on human experience. Purposive and snowball sampling were used to recruit participants. An initial target of approximately 20 participants was set to ensure sufficient depth and variability of experiences. Data adequacy was determined during analysis, with saturation reached when no new themes emerged.

**Inclusion criteria:** Participants were included if they were registered RA pharmacists, based in Johannesburg, Gauteng, and currently working remotely or in a hybrid model. Participants were required to be at least 6 months post-easing of COVID-19 restrictions, registered with the South African Pharmacy Council (SAPC), and willing to participate in an online semi-structured interview

**Exclusion criteria:** Pharmacists who were not practising in RA, who were working exclusively on-site, who were based outside Gauteng or who were not registered with the SAPC were excluded from the study.

### Data collection

The researcher piloted the interview guide in December 2023 to ensure the validity of the study methodology and tools, assess its understandability among participants, and alignment with study objectives. Two RA pharmacists meeting inclusion criteria participated in the pilot interviews. Their data were not included in the final analysis.

The researchers conducted and audio-recorded semi-structured interviews independently in English via Microsoft Teams for Mac (Version 24180.210.3001.6635) (One Microsoft Way, Redmond, WA, United States) took field notes, and conducted each interview for approximately 30 min. Before data collection, participants received the study information sheet and provided written consent for interview participation and voice recording. The researcher reiterated voluntary participation and the right to withdraw at any time without penalty.

Semi-structured interviews were chosen over focus groups and surveys as the method allows for greater flexibility, giving participants the chance to express personal views on sensitive topics such as WLB and well-being while still allowing the researcher to focus on key themes. Thus, enabling the receipt of detailed responses and thick descriptions from study participants.

All interviews were conducted online via Microsoft Teams. No in-person interviews were conducted.

Microsoft Teams proved to be a convenient tool for virtual data collection, offering participants the flexibility to join from any location. Additionally, the platform facilitated the downloading and storage of interview recordings, along with auto-generated transcriptions to the cloud. Technical issues occasionally emerged due to poor bandwidth from load shedding, briefly disconnecting participants mid-interview. The researchers addressed these disruptions by repeating questions and prompting participants to rephrase their answers. When participants were unavailable owing to load shedding, the researchers adapted to their schedules. Member checking took place during the interviews, with the researchers relaying participants’ responses to confirm their understanding and ensure accurate reflection of their perspectives.

### Data analysis

A multi-stage approach for thematic analysis as described by Gale et al. (2013) was used. The Work–Life Harmony and Socioecological frameworks guided deductive coding by demonstrating the influence and associations between individuals and their environments. The analysis comprised transcription, familiarisation with the interviews, coding, developing a working analytic framework, applying a working analytical framework, charting the data into a framework matrix, and interpretation. The transcription feature on Microsoft Teams was used to transcribe the interviews verbatim. The researcher familiarised himself with the data by repeatedly listening to the interview recordings and reviewing interview notes. Transcripts were manually reviewed, formatted, edited for correctness, and assigned unique numerical identifiers for anonymity purposes. These were imported into MAXQDA (2020) for manual and computer-aided coding. MAXQDA (VERBI Software GmbH, Berlin, Germany) is a validated tool for qualitative research data collection, coding, systematic review and thematic analysis (Hart & Achterman 2017). The dataset was labelled with codes by two coders, and recurring codes were grouped into subthemes and refined into themes that corresponded to each layer of the socio-ecological framework. The interviews guided inductive coding, and the overarching themes were categorised according to the socio-ecological framework. These were reviewed iteratively by the team to establish agreement and ensure reliability.

### Trustworthiness

Measures of Trustworthiness are credibility, transferability, dependability and confirmability (Shenton 2004). Credibility was ensured through member checking throughout the research process. The researcher reflected on their positionality as an RA Pharmacist and utilised their expertise to develop a broad understanding of the research setting. Reflexivity was ensured through ongoing self-reflection, the use of open-ended questioning and critical engagement with co-authors during data analysis to minimise the influence of the researcher’s assumptions on data interpretation.

Confirmability was achieved by minimising researcher bias in the data through an inter-coding agreement between co-authors and a qualitative research expert, ensuring alignment of the codes with the study objectives and the conceptual and theoretical framework. Dependability was established by ensuring stability in the data over time, and the Consolidated Criteria for Reporting Qualitative Research (COREQ) checklist was followed. The researcher maintained an audit trail by saving raw data, corrected transcripts and the participants’ consent forms on the cloud drive. Transferability was ensured using thick and comprehensive descriptions (Ahmed 2024) of the study site’s socio-economic and geographical context, participants’ unique work context, data collection considerations and the robust methodology applied.

### Ethical considerations

Ethical clearance to conduct this study was obtained from the University of the Witwatersrand Human Research Ethics Committee (Medical) (No. M230741). Participation in the study was voluntary; participants did not receive compensation or incentives. The researcher obtained written informed consent for interview participation and voice recording prior to data collection. Privacy and confidentiality were protected through the use of unique numerical identifiers, de-identification of transcripts, and secure storage of study materials with access restricted to the researchers. The principle of beneficence was upheld by minimising potential harm during interviews and allowing participants to pause or discontinue participation if discomfort arose. Justice was maintained by applying the eligibility criteria consistently and treating all participants equitably throughout recruitment and data collection. The questionnaire and codes were reviewed by co-authors to ensure there was no researcher bias.

## Findings

### Participant demographics

The study sample included 17 participants: 2 men (11.76%) and 15 women (88.24%). Participants included both parents and non-parents. Parents had one to three children across various developmental stages, ranging from infancy to adolescence. Most participants worked under a hybrid working model, although policies on flexibility varied and changed over time.

An overview of participant demographics is highlighted in Table 1.

**TABLE 1:** Participant demographics and work arrangement.

Demographics	Number of participants
**Sex**	
Male	2
Female	15
**Household arrangement**	
Living with family	13
Living alone	4
Parents	11
Non-parents	6
**Work arrangement**	
Fully remote	1
Hybrid	16
**Roles**	
Managerial role	4
Non-managerial roles	13
**Years of experience in RA (Range)**	
0–4	7
4–10	3
10–20+	7

RA, regulatory affairs.

Participants represented different career stages, with experience ranging from a few months to 20 years. One newly appointed manager stated:

‘Transitioning into a managerial role felt like I needed to be online more, especially due to the international nature of our business and being appointed under dual roles. With time, I realised that constantly being online is psychologically limiting and does not equate to being productive.’ (P15, Female, RA Manager)

This account suggests that WLB was influenced by experience and role transition.

An overview of Themes grouped under barriers and enablers, mapped onto Bronfenbrenner’s framework, is outlined in Table 2 and Online Appendix Table 1-A1.

**TABLE 2:** Barriers and enablers to work–life balance among regulatory affairs pharmacists working remotely, aligned with Bronfenbrenner’s socio-ecological levels.

Bronfenbrenner level	Barriers (consolidated themes	Enablers (consolidated themes)
Microsystem	Individual behaviours and structuring work (Individual level – Microsystem). Isolation and reduced stimulation (for some).	Personal boundary setting and routines.
Mesosystem	Work–family role interference (competing home and work demands; difficulty ‘switching off’).	Family support and work–family enrichment (caregiving involvement; bonding; shared responsibilities).
Exosystem	Managerial control and inconsistent policy implementation (micromanagement; reduced flexibility; monitoring). Organisational readiness and resourcing gaps (inadequate onboarding; limited staffing; insufficient tools or ergonomic support).	Supportive management and peer collaboration (autonomy-supportive supervisors; knowledge sharing).
Macrosystem	Regulatory demands and industry expectations (public health pressures; output-driven culture; cross-territory demands or time zones).	Legitimisation of remote work (evidence of productivity during COVID-19; shifting norms).
Chronosystem	Evolving remote-work arrangements over time (policy shifts post-easing; changes in expectations and availability).	Adaptation over time through learning and rule-setting (development of stricter boundaries and routines).

COVID-19, coronavirus disease 2019.

### Participant definitions of remote work

Participants defined remote work as work performed outside the formal office, most commonly from home but also from locations such as coffee shops. Although remote work offered flexibility, it also blurred boundaries between work and home life, indicating both work-life enrichment and WLC:

‘So, working remotely would be working in your home or like basically not commuting and travelling to the office … I think one of the challenges that I do find working from home is that sometimes your home environment and your work environment is the same. So, if you don’t have like a clear boundary between the two, it can sometimes become like the line is lost.’ (P2, Female, RA Pharmacist)

### Participant perspectives of work-life balance

Participants defined WLB as maintaining boundaries between work and personal roles. Remote work supported family presence and flexibility, indicating work-life enrichment, but also made disengagement from work difficult, contributing to WLC:

‘I think what happens is when it’s time to shut down and you’ve worked in your hours; it doesn’t happen because you’re at home where you don’t need to shut down to catch your transport or leave the office to get home … So, you’ll end up working a bit longer and sometimes very long … The shutting down for me in an ideal way is shut down the computer, you don’t go back to anything regarding work … It’s literally saying I’m done and I concentrate on my family. That for me is a shutdown, remote work has sort of disrupted that … Sometimes I can log into a call at 8 ’o clock at night … It’s past your working hours that you actually working, so there are pros and cons to remote working.’ (P1, Female, RA Pharmacist)

‘Work-life balance for me is when I’m at home, I need to be doing my home relates functions and responsibilities, I need to look after my kids. I need to make sure they are eating, running of the household and when I’m at work forget about my kinds. I need to do what is work-related, so when you are working from home there is an overlap or there is no clear separation. There is no clear cut-off … I find myself when I’m working from home working more hours sitting the whole day, going beyond what I would have normally done if I was at the office … you wake up early, eat, and you’re sitting in front of your computer.’ (P12, Female, RA Pharmacist)

Thematic analysis identified two overarching dimensions, namely WLC and work–life enrichment, organised within Bronfenbrenner’s socio-ecological model.

### Barriers to work–life balance

#### Individual behaviours and structuring work (individual level – microsystem)

Workaholism, perfectionism and difficulty structuring the working day were identified as barriers to WLB. Limited prior experience with remote work and blurred home-work boundaries reduced concentration and extended working hours, indicating WLC:

‘I think for me that was the only issue initially was finding how to actually structure my day while working from home … So how do I structure being at home and being productive throughout the day? … I guess I think it took some time for me to fully work and understand to fully have a good system of working at home efficiently as opposed to being in the office and vice versa … I think what sometimes prohibits me from having a good work life balance is I think finding ways to fully focus, especially looking at working from home.’ (P9, Male, RA Pharmacist)

Participants further indicated that the absence of commuting extended available work time.

Time saved from commuting was often redirected to additional work rather than rest, household responsibilities or self-care. This reinforced overwork and reduced the work-life enrichment associated with flexibility.

#### Isolation and living arrangements (microsystem)

Living arrangements shaped participants’ experiences differently. For some, living alone had little effect on WLB when social connections were maintained. For others, limited human interaction contributed to poor mental health, social disconnection and longer working hours, indicating WLC:

‘It’s become so difficult event to be reaching out because you find that I mean, as I am saying people have lives … I mean it’s Friday, and as I was saying I was going to the gym, to the steam room, buy supper along the way. Switch on my laptop and continue from where I left up until I sleep, because I don’t think they are available.’ (P2, Female, RA Pharmacist)

‘… Living alone and working from home have negatively impacted my depression … I have never been in a situation where I find myself being in now whereby I am kind of learning new ways of surviving and without having that human interaction that is constantly there … What conversations do you have outside of your workplace, do they build you? I feel like I’m diminishing, actually, like my language is also getting minimal because what I talk about mostly is work.’ (P2, Female, RA Pharmacist)

#### Management practices and workload (microsystem and mesosystem)

Management practices shaped WLB at micro- and mesosystem levels. Supportive managers enabled autonomy and flexibility, whereas micromanagement, fixed schedules, after-hours meetings and rising workloads intensified WLC:

‘My supervisor respects our autonomy, but upper management’s micromanaging undermines flexibility.’ (P6, Female, RA Pharmacist)

‘So, we are supposed to give one specific time and then stick to that particular time the whole year. You don’t have the flexibility of today starting at this time ….’ (P6, Female, RA Pharmacist)

‘It’s not that somebody is forcing you, but because of the pressures that we are facing with work, I think it feels like there has been this exponential growth, just the amount of work that needs to be done. You feel like let me compensate by at least working an extra hour in extra hour, which becomes extra four hours working and not spending time with the family.’ (P8, Female, RA Pharmacist)

‘I also felt like there were too many expectations that have arisen because of working from home whereby you know you could be in a meeting even at an inconvenient time.’ (P2, Female, RA Pharmacist)

#### Nature of regulatory affairs work and public health pressures (macrosystem)

Regulatory affairs work was described as demanding and time-sensitive, frequently extending into evenings, weekends and public holidays. Public health responsibilities, accelerated approvals and strict authority timelines intensified WLC:

‘We work with a Health Authority, so there were instances where I would work long hours just to make sure I’m able to keep up to deadlines.’ (P11, Male, RA Pharmacist)

‘There are instances where there will be a spike in terms of the time that you need to be online because health agencies or projects that are short-term have limited time for us to meet those targets.’ (P15, Female, RA Manager)

Some participants accepted these demands because of the perceived public health importance of their work:

‘At that particular point in time, it was COVID, and the work we do is in the interest of public health, so I tend to prioritise it, but it wasn’t that there was a lot of external pressure from anyone to work those long hours.’ (P10, Female, RA Pharmacist)

#### Industry culture and regulatory complexity (macrosystem)

An output-driven industry culture was characterised by urgency, high workload and pressure to deliver. Working across time zones and navigating different regulatory requirements across countries further intensified WLC:

‘So, there is that, but I swear to you, it is so busy … It’s all about putting down the fires. So, umm, you know when you’re putting down the fires you get tired, you get tired because it.’ (P2, Female, RA Pharmacist)

‘The industry that we’re working in is very output driven, so I believe that when they conduct interviews, they’re looking for people who are results driven as well.’ (P4, Female, RA Pharmacist)

‘So, you end up working overtime, for example, just to be able to update systems and other activities that are related to the submissions for the number of countries that I was responsible for.’ (P14, Female, RA Pharmacist)

#### Organisational readiness and onboarding (exosystem)

Organisational policies and readiness shaped WLB. Although remote work policies were generally viewed positively, inconsistent implementation, inadequate tools, poor ergonomics and insufficient onboarding limited their benefits and increased WLC:

‘I find that we’re not capacitated to work from home in this particular space or this particular company, even though we are allowed to … So, it’s simple things like for example, the servers are too slow if you were to work from home or they will not give you an additional screen. With the type of work we do in RA you need a second screen, so that’s a cost you need to incur yourself if you want to work from home … I joined the company as an RA Pharmacist with no prior experience and my manager resigned one month into my joining the company. There was no replacement for them until after three months, and I had to take on that responsibility. This was a challenging transition for me and it impacted my work-life balance negatively as I was the only person in the company working in regulatory affairs. I suffered a seizure for the first time as a result of the stress.’ (P10, Female, RA Pharmacist)

Adequate onboarding, training and staffing were identified as necessary for business continuity and sustainable work practices. Limited staffing and uneven workload distribution increased strain and reduced well-being:

‘The expectation is that you’ll be able to deliver no matter what, and you can’t even talk to them about it. I told management that we needed to appoint more personnel, but I ended up taking the weight. Although I had a team, the team was not sufficient enough to carry the load. So, I ended up being operational as well, and that resulted in long hours of working to such an extent that I was not becoming functional at all anymore because I was forever tired. When you are tired, you end up making mistakes; you may also even make grave mistakes that will cost the company even more, and that may even affect people’s lives in a way.’ (P15, Female, RA Manager)

These accounts suggest that flat structures and limited human resources shifted additional responsibilities to individual employees, undermining WLB.

Participants also described an organisational culture that equated overtime with productivity, further intensifying WLC:

‘There’s this notion that working overtime equates to productivity, but that’s not true … I’ve been in this industry for a while and I have had a bad view of South African companies especially. They really have a bad view of how remote work is and how being a manager or what being a manager is and even being employees, you’re expected to work 24/7. We celebrate those employees that work 100 hours a week or 200 hours a week.’ (P15, Female, RA Manager)

### Enablers to work–life balance

#### Boundary setting and routines (individual level – microsystem)

Boundary setting and routine formation were identified as enablers of work-life enrichment. Physical and temporal separation between work and home, regular breaks, communication of household expectations and planning across time zones supported productivity and well-being:

‘I start my day as if I’m going to the office, with breaks and a set end time to shut down work.’ (P14, Female, RA Pharmacist)

‘I had to consciously dedicate myself fully to a task within a specific timeframe. When that time is up, I redirect my energy and give my all to the next task. At the same time, I prioritise my time, making space for quiet reflection, personal activities, and passions. This might include tending to your garden, reading, taking walks, or engaging in community activities. I believe these practices are essential for building psychological and emotional well-being.’ (P15, Female, RA Manager)

#### Family dynamics and support (microsystem)

Family support was identified as a key facilitator of work-life enrichment. Spouses, parents, siblings, children and helpers assisted participants to manage household responsibilities, maintain boundaries and prioritise family time:

‘I wouldn’t have been able to cope in my new role without my husband. My siblings and parents have also played a role as we can share tasks such as picking up kids and doing homework.’ (P14, Female, RA Pharmacist)

Clear communication within the household also reduced interruptions and supported remote work functioning.

‘So, it’s important to communicate even with your 5-year old and it’s amazing how kids have adapted also in this time … So specifically, she knows that if Mommy’s in the meeting, I also need to respect that.’ (P1, Female, RA Pharmacist)

#### Family bonding and caregiving (mesosystem)

Remote work promoted work-life enrichment by creating opportunities for caregiving, family bonding and greater involvement in children’s development:

‘In terms of the Family part I regard it as a huge benefit and a privilege actually to be able to work remotely, simply because I don’t have any other support system beside myself and my spouse. So, the responsibility for my two children would rely fully on my husband and myself, so the remote working has hugely assisted being able to be there for my children at times when I wouldn’t normally be able to be there if I was working fully in the office, and that has actually helped even engagement with my children and I think it’s built a far more almost very trusting and appreciative relationship with my children. So it’s not just about the relationship that it’s enabled with them, but they also can see the benefit of me being available to them where I wasn’t previously.’ (P13, Female, RA Manager)

#### Peer support (workplace interactions – microsystem and mesosystem)

Peer support enabled work-life enrichment through knowledge sharing, practical guidance and mutual encouragement in managing workplace demands:

‘We help each other survive the workplace through knowledge sharing and mutual encouragement.’ (P1, Female, RA Pharmacist)

## Discussion

This discussion is structured in line with the study findings and Bronfenbrenner’s socio-ecological framework.

It highlights how barriers and enablers to WLB operate at, and interact across, individual, relational, organisational and broader contextual levels. This study therefore explores the barriers and enablers influencing WLB among RA pharmacists working remotely in Gauteng. Overall, remote and hybrid work afforded flexibility and opportunities for work–family enrichment. These benefits were often offset by WLC linked to boundary erosion, extended availability and workload pressures.

### Microsystem: Individual behaviours, boundary management and isolation

At the individual level, participants described how remote work reduced commuting time and created perceived ‘extra time’. This time was often reallocated to additional work, which contributed to self-imposed extended hours and difficulty ‘shutting down’. Similar patterns have been reported in qualitative studies of remote work, where the removed physical boundary between work and home can intensify WLC and overwork (Adisa et al. 2022; Taksi Deveciyan & Alay 2023). Participants also described an initial adjustment period as they learnt to structure the workday when working from home. This highlights the importance of routines and boundary-setting as proximal enablers of WLB. These findings can be interpreted through boundary and work–family border theory, which suggests that individuals actively construct, maintain and negotiate boundaries between work and non-work roles to minimise role conflict, and that when these boundaries are weakened or blurred, greater work–life interference and difficulty disengaging from work may occur (Ashforth, Kreiner & Fugate 2000; Clark 2000).

For some participants living alone, reduced social interaction was linked to poorer well-being. This finding aligns with O’Hare et al. (2024), who reported that healthcare workers who spent more hours working remotely experienced higher stress and provides evidence that household composition may shape remote workers’ support needs and mental health outcomes (Kangas et al. 2023).

### Mesosystem: Work–family role interference and enrichment

Consistent with the Work–Life Harmony model, participants’ accounts reflected both work–family conflict and work–family enrichment (Hasan et al. 2020). Remote and hybrid work enabled greater presence in family life and caregiving involvement, particularly for parents and those with limited support systems. However, proximity also created competing demands. Household responsibilities sometimes intersected with work requirements and extended the working day. Similar mixed effects have been reported in broader remote working literature, where remote work can support family involvement while also increasing role interference for some households (Harry Williamson – Labour Market and Skills Unit 2022; Kangas et al. 2023).

### Exosystem: Organisational policy, managerial practices and resourcing

At the organisational level, participants emphasised that managerial practices and policy implementation shaped whether remote work functioned as an enabler or a barrier. Supportive supervisors facilitated autonomy. In contrast, micromanagement and monitoring undermined flexibility and contributed to extended availability. This supports earlier work showing that flexibility is not only about location, but also about how work is managed and controlled (Allen et al. 2013, 2015). Participants also reported uneven application of hybrid policies. This created uncertainty and reduced perceived fairness. Organisational readiness constraints further intensified workload. These included limited staffing, inadequate onboarding and insufficient tools or ergonomic support. In this context, remote work may shift costs and burdens to employees, which can undermine well-being and sustainable performance; this is consistent with broader evidence on home-based work (Berg, Humblet & Soares 2021).

### Macrosystem: Regulatory context, industry culture and societal expectations

The macrosystem context of regulatory work and public health imperatives was central to participants’ descriptions of workload, time pressure and role prioritisation. Working across multiple territories and time zones, and meeting stringent regulatory authority and internal timelines, normalised after-hours work and weekend labour. Participants also described an output-driven industry culture that valorised overtime. This resonates with earlier evidence that telecommuting can offer flexibility, but may not reduce work pressure when job demands remain high (Allen et al. 2015; Gajendran & Harrison 2007). Notably, participants acknowledged that COVID-19 demonstrated the feasibility of remote work and sustained productivity. However, this may also contribute to heightened organisational expectations if productivity is pursued without regard for sustainability and well-being (Oleksa-Marewska & Tokar 2022).

### Chronosystem: Evolving remote-work arrangements over time

A time-based perspective is important when interpreting participants’ experiences following the easing of COVID-19 restrictions. Participants described changes in policy strictness and increased managerial discretion over time, reflecting broader post-pandemic policy shifts as organisations moved from emergency remote work arrangements to more formalised hybrid models. This finding aligns with broader evidence presented by Vyas (2022), demonstrating that the post-pandemic period is characterised by a structural transition from crisis-driven remote work to more formalised and institutionalised hybrid working arrangements. Participants further described personal learning curves as they developed routines and boundaries. This suggests that WLB in remote and hybrid arrangements is not static. It is shaped by ongoing adaptation at individual and organisational levels. This reflects evidence presented by Vyas (2022), demonstrating that hybrid working arrangements in the post-pandemic period are characterised by ongoing organisational adaptation as systems transition from crisis-driven remote work to more structured models. The normalisation of remote work further reinforced its legitimacy as a sustainable way of working, although it also appeared to intensify expectations around flexibility, responsiveness and sustained productivity. As remote work becomes normalised, regular policy review and manager capability building remain important to support work–life harmony and limit the re-emergence of constant availability norms (Hasan et al. 2020).

### Cross-domain interactions and implications

Across levels, the findings illustrate cross-domain effects. Cross-domain interactions showed how WLC and work–life enrichment operated across the socio-ecological levels (Hasan et al. 2020; Online Appendix Table 1-A1 and Figure 1-A1). Organisational expectations and regulatory pressures shaped individual boundary practices and family interactions, and vice versa (Hasan et al. 2020). Work–life harmony was more evident when supportive relationships and clear routines co-existed with autonomy-supportive management and adequate resourcing. In contrast, micromanagement, staffing constraints and an overtime-oriented culture interacted with blurred home–work boundaries. This intensified WLC and undermined well-being. Similar interaction patterns have been described in qualitative research on boundary management in remote work (Adisa et al. 2022). These findings support the need for multi-level interventions. These interventions should combine individual boundary management with clear organisational policies, adequate resourcing and leadership practices that prioritise sustainable performance.

### Recommendations

#### Policy recommendations for policymakers

National remote-work guidelines are needed to standardise remote and hybrid work arrangements across sectors. These should outline working hours, mandatory breaks, communication norms and written remote-work policies agreed upon by employees and managers, while allowing reasonable autonomy in the selection of remote and in-office days within operational requirements.

Support for mental health and well-being should be strengthened through employee assistance programmes, stress-management resources, counselling services and workplace wellness initiatives that address burnout in remote work settings. Equally important is investment in digital infrastructure, including reliable internet access, technology, appropriate home-office support and staff training to improve digital literacy and the effective use of artificial intelligence (AI) in remote work. Training for managers on effective remote team management should also be encouraged, with dedicated modules on mental health awareness, flexible leadership and inclusive management practices, while emphasising outcomes-based supervision rather than micromanagement.

#### Reforms to South African Labour Law

A legislated right to disconnect should be considered to protect employees from responding to work-related communications outside ordinary working hours, except in emergencies. This reform should be accompanied by work-hour monitoring mechanisms. Employers should therefore be required to record and report overtime, compensate employees for additional hours worked and ensure that weekly working hours comply with Chapter Two, ‘Regulations of working time’, sections 2 to 18 of the *Basic Conditions of Employment Act* (BCEA).

Stronger protections for remote workers may be achieved through amendments to the BCEA that address workplace safety and ergonomic conditions in home-office environments. Regular review of work arrangements, together with employee consultation when remote-work practices are amended, would help ensure alignment with employee needs and legal standards. Expanded parental and caregiver support, including shared parental leave and flexible caregiving provisions, may further promote WLB.

#### Limitations

The study’s geographic focus was narrow, which may limit the transferability of its results. Gauteng is a densely populated urban province, and some findings might be specific to this locality. Future studies could benefit from multi-provincial sampling and segmentation across various sectors of RA, such as Medical Devices, Veterinary and Complementary Medicine, to enhance the transferability of findings or explore diverse experiences. Additionally, the underrepresentation of male perspectives could warrant a follow-up focus group study with more male participants.

## Conclusion

Findings indicate that remote and hybrid work afforded RA pharmacists flexibility, improved family engagement and reduced commuting, yet frequently intensified WLC through blurred boundaries, overtime and norms of extended availability. Micromanagement, inconsistent policy application and structural constraints, including staffing shortages, inadequate onboarding and limited ergonomic and tool support, further undermined autonomy and were, at times, associated with unfavourable mental health outcomes, particularly among those living alone. Recommendations therefore emphasise standardised remote-work guidelines, investment in digital infrastructure, strengthened mental health support through employee assistance programmes and manager training that prioritises outcomes over monitoring. In addition, the authors propose policy measures to strengthen boundary management and worker protections, including a right to disconnect, overtime monitoring and compensation, ergonomic protections, regular policy review, and enhanced parental and caregiver support.
